# A graph-based filtering method for top-down mass spectral identification

**DOI:** 10.1186/s12864-018-5026-x

**Published:** 2018-09-24

**Authors:** Runmin Yang, Daming Zhu

**Affiliations:** School of Computer Science and Technology, Shandong University, 1500, Shun Hua Lu, Jinan, 250101 China

**Keywords:** Mass spectrometry, Filtering algorithm, Spectrum graph

## Abstract

**Background:**

Database search has been the main approach for proteoform identification by top-down tandem mass spectrometry. However, when the target proteoform that produced the spectrum contains post-translational modifications (PTMs) and/or mutations, it is quite time consuming to align a query spectrum against all protein sequences without any PTMs and mutations in a large database. Consequently, it is essential to develop efficient and sensitive filtering algorithms for speeding up database search.

**Results:**

In this paper, we propose a spectrum graph matching (SGM) based protein sequence filtering method for top-down mass spectral identification. It uses the subspectra of a query spectrum to generate spectrum graphs and searches them against a protein database to report the best candidates. As the sequence tag and gaped tag approaches need the preprocessing step to extract and select tags, the SGM filtering method circumvents this preprocessing step, thus simplifying data processing. We evaluated the filtration efficiency of the SGM filtering method with various parameter settings on an *Escherichia coli* top-down mass spectrometry data set and compared the performances of the SGM filtering method and two tag-based filtering methods on a data set of MCF-7 cells.

**Conclusions:**

Experimental results on the data sets show that the SGM filtering method achieves high sensitivity in protein sequence filtration. When coupled with a spectral alignment algorithm, the SGM filtering method significantly increases the number of identified proteoform spectrum-matches compared with the tag-based methods in top-down mass spectrometry data analysis.

**Electronic supplementary material:**

The online version of this article (10.1186/s12864-018-5026-x) contains supplementary material, which is available to authorized users.

## Background

Top-down mass spectrometry (MS) is an important technology for identifying proteoforms with primary sequence alterations, such as post-translational modifications (PTMs) and mutations [1], because it provides “a bird’s eye” view of whole proteoforms. Reliable identification of protein alterations plays an important role in understanding biological mechanisms underlying diseases [2].

Represented by tools such as ProsightPC [3, 4] and TopPIC [5], database search [6, 7] is the dominant approach for top-down MS-based proteoform identification, in which top-down tandem mass (MS/MS) spectra are searched against a protein sequence database to identify the best matches between the spectra and protein sequences in the database. Maping a query spectrum generated from a proteoform with multiple alterations to its corresponding database protein sequence without alterations is a challenging computational problem.

Methods for identifying proteoforms with alterations are classified into two approaches. The first approach is to use reported protein alterations in the literature to build an extended proteoform database [4, 8]. For example, phosphorylation, acetylation, ubiquitylation and sumoylation sites have been reported in the tumor protein p53 [9], and the knowledge can be used to build a proteoform database of the protein. Using an extended database simplifies database search if query spectra are generated from proteoforms with known alterations in the database. However, there are its limitations in extended database approach. Many PTM sites of proteins are unknown because the proteins have not been extensively studied. Even if all PTM sites of a protein are known, the number of combinations of these PTM sites is an exponential function of the number of sites because each PTM site may or may not be present in a proteoform. As a result, it is impractical to construct a complete extended protein database.

The second approach uses spectral alignment algorithms [5–7, 10–12] to align top-down MS/MS spectra from modified proteoforms against unmodified database protein sequences. Although spectral alignment is effective for single protein spectrum matches, it is very time consuming to align thousands of query spectra to against thousands of protein sequences in a large database. Therefore, efficient protein sequence filtering algorithms are critical for accelerating database search in proteome-level proteomics research [6].

For a gene or a transcript, most protein databases, such as RefSeq [13] and UniProt [14], contain only one unmodified reference sequence. Given a query MS/MS spectrum and a database of unmodified protein sequences, the object of a protein sequence filtering algorithm is to quickly filter out most unmatched protein sequences in the database while keeping the target one that produced the query spectrum. Fragment masses of the query spectrum is more important than the precursor mass for protein sequence filtration because the precursor mass does not match the molecular mass of its corresponding database protein sequence when the target proteoform contains alterations.

In bottom-up and top-down MS [15–18], many filtering methods have been proposed. A popular filtering approach is to use sequence tags, which are partial protein sequences extracted from mass spectra, to scan and filter protein sequences [15–17]. A sequence tag extracted from a query spectrum is correct if it is a substring of the protein sequence that generated the spectrum. Long correct sequence tags are extremely useful in protein sequence filtration because the chance that a long correct tag is matched to a non-target protein sequence is low, but it’s difficult to extract long correct tags from mass spectra due to missing peaks.

To solve this problem, Jeong et al. introduced gapped tags that can be extracted from spectra with missing peaks [19]. Many MS/MS spectra do not contain correct long sequence tags but contain correct long gapped tags. The introduction of gapped tags gave rise to the blocked pattern matching problem, in which a gapped tag is searched against a text string to find matched substrings [20, 21]. It is challenging to distinguish signal peaks from noise ones due to the complexity of MS/MS spectra. Even though the gapped tag approach solves the problem of missing peaks in tag extraction, it is still highly possible that many incorrect tags are reported because of noise peaks.

This paper is an extension of the work originally reported in [22]. In this paper, we propose the spectrum graph matching (SGM) problem and a novel filtering algorithm based on SGM, which constructs spectrum graphs from subspectra of query spectra, and uses the spectrum graphs to filter protein sequences. To our best knowledge, we are the first to study the SGM problem. The SGM filtering method simplifies protein sequence filtration by skipping tag extraction and selection steps. It directly searches spectrum graphs generated from subspectra with noise peaks against protein sequences. A suffix tree based algorithm was proposed in [21] for searching gapped tags against a text string. Our main contribution in method development is to extend the algorithm in [21] for searching spectrum graphs against a protein database. Experimental results on real top-down mass spectrometry data demonstrated that the SGM filtering method achieved high efficiency in protein sequence filtration.

## Methods

The top-down MS/MS spectrum of a proteoform consists of a precursor mass and a list of fragment ion peaks. The precursor mass corresponds to the molecular mass of the proteoform and the fragment ion peaks correspond to fragments of the proteoform. Because of the existence of highly charged fragment ions and isotopic peaks, top-down MS/MS spectra are usually very complex. In data preprocessing, spectral deconvolution tools [23, 24] are used to converted fragment ion peaks into monoisotopic fragment masses. The intensity of a monoisotopic mass is computed as the sum of the intensities of its corresponding fragment ions peaks.

Noise masses need to be removed from deconvoluted top-down MS/MS spectra to improve the accuracy of protein sequence filtering. Generally speaking, low-intensity masses are more possible to be noise ones than high-intensity masses. An intensity-based method is used to remove noise masses from a deconvoluted spectrum. For each mass *x* in the spectrum, we rank all the masses in the interval [*x*−100,*x*+100] with a width of 200 Dalton (Da) in the decreasing order of their intensities. If *x* is not one of the top *λ* masses, *x* is treated as a noise mass and removed, where *λ* is a user-specified parameter. All parameters used in the SGM filtering algorithm are summarized in Additional file 1.

### Spectrum graphs

**Fig. 1:**
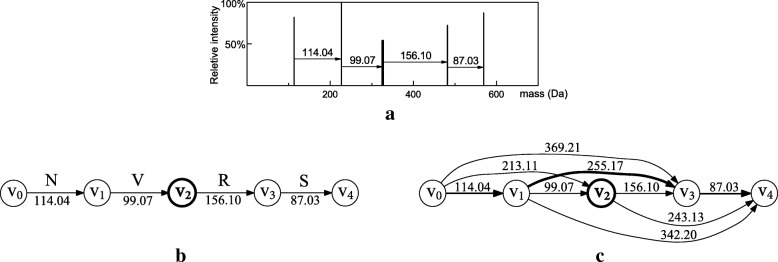
Spectrum graph generation. Illustration of spectrum graph generation using an example deconvoluted MS/MS spectrum of the protein LNRVSG. **a** In the spectrum, the mass of the N-terminal fragment LNR is missing, and there is a noise mass peak (bold) between the fragment masses of LNR and LNRV. **b** In the spectrum graph, each node corresponds to a peak in the spectrum. Two nodes are connected by a directed edge if the difference between their corresponding masses matches the residue mass of one amino acid; the edge is labeled with the amino acid. The sequence tag NVRS extracted from the spectrum is incorrect because of the noise mass peak and its node *v*_2_. **c** In the spectrum graph, each node corresponds to a peak in the spectrum. Two nodes are connected by a directed edge if the difference between their corresponding masses is less than 400 Da and matches the residue mass of one or several amino acids; the edge is labeled by the mass difference. The mass sequence of a path is a blocked pattern of the spectrum. For example, the bold path *v*_0_,*v*_1_,*v*_3_,*v*_4_ corresponds to a blocked pattern 114.04, 255.17, 87.03, which matches a correct sequence tag NRVS because 255.07 is the sum of the mass 156.10 of R and the mass 99.07 of V

Spectrum graphs have been used in sequence tag-based protein sequence filtration to extract sequence tags from mass spectra [25]. There are two steps to generate a spectrum graph from a deconvoluted query spectrum: (a) a node is added to the graph for each fragment mass in the spectrum, and (b) a directed edge from a node *u* to a node *v* is added to the graph if mass(*v*)−mass(*u*) matches the mass of one amino acid residue within an error tolerance, where mass(*u*) and mass(*v*) are the masses corresponding to *u* and *v*, respectively. The edge is labeled with the amino acid explaining the mass difference (Fig. 1b). Each path in a spectrum graph corresponds to a sequence tag, which is the readout of the labeled amino acids on its edges. For example, the only path from node *v*_0_ to *v*_4_ in the spectrum graph in Fig. 1b corresponds to the sequence tag NVRS. There are many existing methods for extracting sequence tags for protein sequence filtration from spectrum graphs [17, 26].

When a spectrum misses many fragment masses, the mass differences of many node pairs in the spectrum graph are explained by 2 or more amino acids, not single amino acids. In this case, the spectrum graph approach described in the previous paragraph may fail to extract long correct sequence tags, which are extremely useful in protein sequence filtration.

Blocked patterns [20] or gapped sequence tags [19] are often used to address the missing peak problem in tag generation. To extract blocked patterns, we change step (b) in spectrum graph generation as follows: a directed edge is added into the graph from a node *u* to a node *v* if the mass difference mass(*v*)−mass(*u*) is explained by a combination of several amino acids and is no larger than a predefined bound *α* (*α* is chosen between 200 and 400 Da in practice). The edge is labeled with the mass difference mass(*v*)−mass(*u*) (Fig. 1c). Each path in a spectrum graph with labeled masses corresponds to a mass sequence, called a *blocked pattern* or a gapped sequence tag [20]. In block pattern-based filtration, multiple blocked patterns (gapped sequence tags) extracted from a spectrum graph are searched against protein sequences to find candidate ones. The number of blocked patterns in a spectrum graph may be an exponential function of the length of the longest blocked pattern, making it inefficient to explicitly extract all blocked patterns in the spectrum graph. As a result, it is common to report only a limited number of paths and their corresponding blocked patterns in a spectrum graph. Because of the existence of noise peaks, it is a challenging problem to determine which paths in a spectrum graph correspond to blocked patterns that match the target protein sequence. We propose to circumvent the blocked pattern selection step and directly search spectrum graphs against protein sequences for filtration.

### The spectrum graph matching problem

For a gene or a transcript, most protein databases contain only one unmodified reference sequence. To simplify the description of the SGM filtering method, we assume that protein databases used in spectral identification contain only unmodified sequences and that amino acid residue masses are integers. All amino acid residue masses are discretized by first multiplying the masses by a scale factor (100 was used in the experiments) and then rounding the results to integers.

Notations introduced by Deng et al. [21] are used in the description of the SGM problem. An amino acid string is represented as a list of discretized residue masses: the mass representation of an amino acid string *a*_1_,*a*_2_,…,*a*_*n*_ is a residue mass string *S*=*s*_1_,*s*_2_,…,*s*_*n*_, in which *s*_*i*_ is the discretized residue mass of *a*_*i*_ for 1≤*i*≤*n*. Residue mass strings are called *text strings* in the following analysis. The sum of all the masses in a substring *s*_*i*_,*s*_*i*+1_,…,*s*_*j*_ of *S*, i.e., $\sum _{k=i}^{j} s_{k}$, is called the *mass of the substring*. Two substrings of *S* are called *consecutive substrings* if the first residue mass of the second string directly follows the last residue mass of the first string. For example, *s*_*i*_,*s*_*i*+1_,…,*s*_*j*_ and *s*_*j*+1_,*s*_*j*+2_,…,*s*_*k*_ are consecutive substrings of *S*. A sequence of consecutive substrings *A*_1_,*A*_2_,…,*A*_*k*_ that include all residue masses in *S* is called a partition of *S*. The masses of the consecutive substrings in a partition is called the mass string of the partition. For example, let *S*=114,156,99,87, *A*_1_=114,*A*_2_=156,99, and *A*_3_=87, then *A*_1_,*A*_2_,*A*_3_ is a partition of *S* and the mass string of the partition is 114,255,87, where 255 is the mass of *A*_2_. A blocked pattern obtained from a path in a spectrum graph is represented by a sequence of masses, which are labels of the edges in the path. For example, the blocked pattern corresponding to the bold path in Fig. 1c is 114.04, 225.17, 87.03. A blocked pattern can be further discretized to an integer sequence using the same method for residue mass discretization. In the following analysis, we assume that all blocked patterns are integer ones. Unlike text strings, a blocked pattern contains not only single amino acid residue masses, but also those of combinations of several amino acids. A blocked pattern *P* matches a text string *S* if *P* is the mass string of a partition of *S*. For example, the blocked pattern 114,255,87 matches the text string 114,156,99,87 because the blocked pattern is the mass string of a partition of the text string.

In protein sequence filtration, we search blocked patterns extracted from a spectrum graph against a protein database to find matched amino acid sequences (sequence tags). All protein sequences in the database are concatenated and represented as a text string over an alphabet *Σ* containing the discretized residue masses of the 20 common amino acids. Because the masses of leucine and isoleucine are the same, the size of *Σ* is 19 instead of 20. Given a text string *S* over an alphabet *Σ* and a blocked pattern *P*, the blocked pattern matching problem is to find all substrings of *S* that match *P*.

#### **Definition 1**

Given a text string *S* over an alphabet *Σ* and a pattern *P*, the blocked pattern matching (BPM) problem is to find all substrings of *S* that match *P*.

The SGM problem is an extension of BPM problem that considering the noises in the spectrum. It is more complex than the blocked pattern matching problem.

#### **Definition 2**

Given a text string *S* over an alphabet *Σ* and a spectrum graph *G*, the spectrum graph matching (SGM) problem is to find all substrings of *S* that match a blocked pattern in *G*.

We first study a restricted version of the SGM problem, in which a start node and an end node in *G* are specified and only paths from the start node to the end node are used to find matched substrings of the text string. The SGM problem is solved by enumerating all node pairs in *G* as the start and end nodes in the restricted spectrum graph matching (RSGM) problem.

#### **Definition 3**

Given a text string *S*, a spectrum graph *G* over an alphabet *Σ*, a start node *s*, and an end node *t*, the restricted spectrum graph matching (RSGM) problem is to find all substrings of *S* that match a blocked pattern corresponding to a path from *s* to *t* in *G*.

### A suffix tree based algorithm for the RSGM problem

To solve the RSGM problem, we present a suffix tree based algorithm that extends the algorithm proposed by Deng et al. for the blocked pattern matching problem [21]. A suffix tree is used to represent the string *S*. We assume that each edge in the suffix tree is labeled with only one letter (integer residue mass) to simplify the algorithm description.

We first review the algorithm for the blocked pattern matching problem, which was proposed by Deng et al. [21]. A blocked pattern is represented as a spectrum graph in which all edges are in one path. Let *G*={*V*,*E*} be the graph representation of a blocked pattern *P*=*p*_1_,*p*_2_,…,*p*_*m*_, where *V*={*v*_0_,*v*_1_…,*v*_*m*_} and *v*_0_,*v*_1_,…,*v*_*m*_ is the only path from *v*_0_ to *v*_*m*_ in *G*. Each prefix *p*_1_,*p*_2_,…,*p*_*k*_ of the blocked pattern *P* corresponds to a path *v*_0_,*v*_1_,…,*v*_*k*_. A text string over *Σ* that matches the prefix *p*_1_,*p*_2_,…,*p*_*k*_ is called a prefix text string of *v*_*k*_. A prefix text string is *identifiable* if it is a substring of *S*. For example, when *P*=114,255,87, both 114,156,99 and 114,99,156 are prefix text string of *P*. When *S*=114,156,99,87, the string 114,156,99 is an identifiable prefix text string of *P*, but 114,99,156 is not. If a prefix text string is not identifiable, then all text strings with the prefix are not identifiable, making it not necessary to search these text strings in *S*. Using identifiable prefix text strings significantly improves in the speed of searching a blocked pattern against *S* represented as a suffix tree.

Let *B*_*i*_ be the set of nodes in the suffix tree corresponding to all identifiable prefix text strings of *v*_*i*_ for 0≤*i*≤*m*, where *m* is the length of the blocked pattern. Specifically, *B*_0_ contains only the root node of the suffix tree. The blocking pattern matching algorithm progressively finds the node sets *B*_1_,…,*B*_*m*_ in the suffix tree. The node set *B*_*m*_ contains the solution to the blocked pattern matching problem.

Unlike the blocked pattern matching problem with only one blocked pattern, the spectrum graph *G* in the RSGM problem contains many paths from the start node to the end node, each path corresponding to a blocked pattern. The number of all paths in a spectrum graph may be an exponential function of the number of nodes. So it is an inefficient approach to directly extract all paths in a spectrum graph and search each path against a suffix tree separately.

Next, we describe our new algorithm for the RSGM problem, which extends the blocked pattern matching algorithm. Let *V*={*v*_0_,*v*_1_,…*v*_*m*_} be the set of nodes in the increasing order of their corresponding masses, in which the start node *s* is *v*_0_ and the end node *t* is *v*_*m*_. A text string is a prefix text string of node *v*_*i*_ if it matches a blocked pattern corresponding to a path from *v*_0_ to *v*_*i*_. Let *B*_*i*_ be the set of nodes in the suffix tree corresponding to all identifiable prefix text strings of *v*_*i*_, and *C*(*e*) be the set of all text strings whose masses match the labeled mass of *e* (Table 1). In practice, a precomputed table is used to quickly obtain *C*(*e*). Because the fragment masses in query spectra have measured errors, an error tolerance *ε* is allowed in generating the text strings in the table. Denote *E*_*i*_⊆*E* the set of all edges whose tail nodes are *v*_*i*_. For each edge *e*=(*v*_*j*_,*v*_*i*_) in *E*_*i*_, we search from each suffix tree node in *B*_*j*_ to find all the paths corresponding to a text string in *C*(*e*) and add the end nodes of these paths to *B*_*i*_ (Algorithm 1).

**Table 1:** An example set of text strings matched a mass 270.14

No.	Amino acid string	No.	Amino acid string
1	QAA	7	GAAA
2	AQA	8	AGAA
3	AAQ	9	AAGA
4	RGG	10	AAAG
5	GRG	11	NR
6	GGR	12	RN

In proteoform identification, there are 20 common types of amino acids. The scaling factor 100 is used for the discretization of the residue masses of the 20 amino acids. The alphabet consists of 19 integers because leucine and isoleucine have the same discretized mass 11308. There are a total of 12 text strings whose descritized masses are 27014. For brevity, all masses in the text strings are represented by their corresponding amino acids. For example, the text string 11404, 15610 is represented by NR


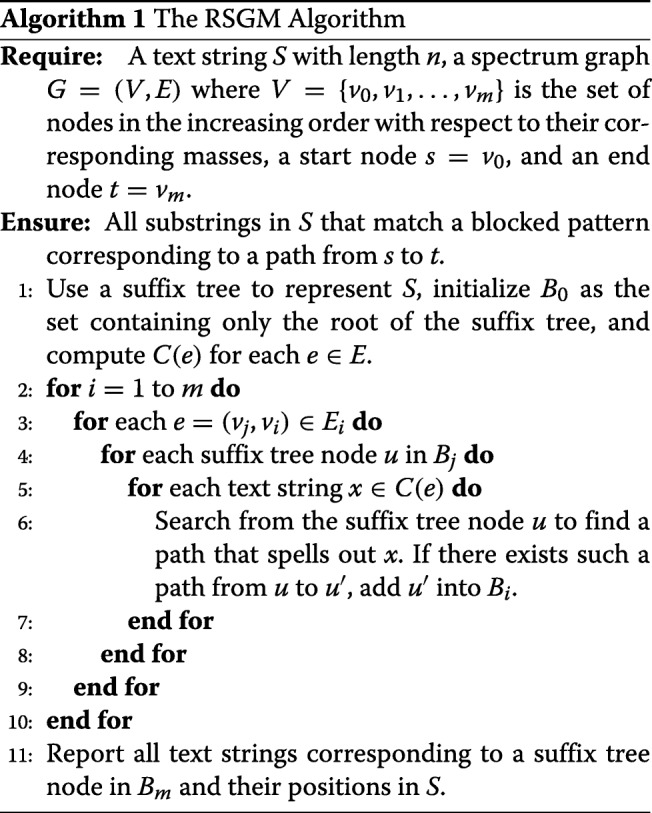


After the last set *B*_*m*_ is found, the RSGM problem is solved by reporting all the text strings corresponding to a suffix tree node in *B*_*m*_ and their positions in *S*, which are stored in the suffix tree. Because the string *S* is represented by a suffix tree, the space complexity and time complexity of the algorithm are both *O*(*n*), where *n* is the length of the string *S*.

### Time complexity

We use the idea proposed by Deng et al. to prove the time complexity of the RSGM algorithm [21]. The preprocessing step, in which the text *S* is represented as a suffix tree, is implemented using the Ukkonen’s algorithm [27], and its time complexity is *O*(*n*). Below we study the time complexity of the pattern query steps in the RSGM algorithm (Steps 2-11 of the algorithm in Algorithm 1).

We divide the set *C*(*e*) of text strings for an edge *e* in the spectrum graph into subsets *C*(*e*,1),*C*(*e*,2), …,*C*(*e*,*l*), where *l* is the length of the longest text string in *C*(*e*). The subset *C*(*e*,*j*) contains all text strings in *C*(*e*) with length *j*. The *expansion factor* of the set *C*(*e*,*j*) is defined as $r(e,j) = |C(e,j)|^{\frac {1} {j}}$, where |*C*(*e*,*j*)| is the size of *C*(*e*,*j*). The largest expansion factor of all edges in *G* is denoted as *r*= max*e*,*jr*(*e*,*j*). Let *N* be the maximum size of *C*(*e*) for all edges in *G*, that is, *N*= max*e*∈*E*|*C*(*e*)|.

The running time of the graph query (Steps 2 - 11) in the RSGM algorithm is related to $\sum _{i=1}^{m}|B_{i}|$, the sum of the sizes of the sets *B*_*i*_. Next we will describe how to compute $\sum _{i=1}^{m} |B_{i}|$. Let *U* be the set of all prefix text strings of *v*_1_,…,*v*_*m*_, and *L* be the length of the longest prefix text string in *U*. We define *X*_*l*_ as the set of all length *l* prefix text strings in *U*, and *Y*_*l*_ as the set of all length *l* identifiable prefix strings in *U*, for 1≤*l*≤*L*. Each node in $\cup _{i=1}^{m}B_{i}$ corresponds to an identifiable prefix text string in $\cup _{l=1}^{L}Y_{l}$, that is, $\sum _{i=1}^{m}|B_{i}| = \sum _{l=1}^{L}|Y_{l}|$. We compute the expectation of |*Y*_*l*_| by multiplying the size of *X*_*l*_ and the probability that a length *l* text string can be found in *S*.

A path in *G* starting from node *s* is called a prefix path. Let *Q* be a prefix path of *G* and *X*_*l*_(*Q*) the set all prefix text strings with length *l* that match a prefix subpath of *Q*. Deng et al. proved the following Lemma about the size of *X*_*l*_(*Q*) [21].

#### **Lemma 1**

The size of *X*_*l*_(*Q*) is bounded by (2*r*)^*l*^, where *r* is the maximum expansion factor.

Based on Lemma 1, we give an upper bound of the size of *X*_*l*_.

#### **Lemma 2**

The size of *X*_*l*_ is bounded by (2*rd*)^*l*^, where *d* is the maximum out-degree of the nodes in *G*.

#### *Proof*

Each length *l* prefix text string in *X*_*l*_ matches at least one prefix path. The total number of prefix paths in *G* with length *l* is bounded by *d*^*l*^. Because of Lemma 1, the number of prefix text strings in *X*_*l*_ matching one prefix path in *G* is bounded by (2*r*)^*l*^. As a result, the size of *X*_*l*_ is bounded by (2*r*)^*l*^*d*^*l*^=(2*rd*)^*l*^. □

Let *g*=|*Σ*| be the size of the alphabet *Σ*. Using the same proof method in Lemmas 1 and 2 in the supplementary material in [21], we can prove the following two lemmas.

#### **Lemma 3**

When 2*rd*<*g*, the expectation of |*Y*_*l*_| is bounded by $\phantom {\dot {i}\!}(2rd)^{{\text {log}_{g}}n}=n^{{\text {log}_{g}}(2rd)}$, where *n* is the length of the text string *S*.

#### **Lemma 4**

When 1<2*rd*<*g*, the expectation of ${\sum \nolimits }_{i=1}^{m}|B_{i}|$ in the RSGM algorithm is bounded by $\phantom {\dot {i}\!}c\,n^{{\text {log}_{g}}(2rd)}$, where $\phantom {\dot {i}\!}c=\frac {2rd}{2rd-1}+\frac {g}{g-2rd}$.

#### **Theorem 1**

When *Σ* is a finite set and 1<2*rd*<*g*, the average-case time complexity of the graph query steps in the RSGM algorithm is *O*(*dNn*^*k*^+*M*), where *d* is the maximum out-degree of the nodes in *G*, *N*= max*e*∈*E*|*C*(*e*)|, *k*= log*g*(2*rd*)<1, and *M* is the number of matched substrings in *S*.

#### *Proof*

For each suffix tree node *u* in $\cup _{i=0}^{m} B_{i}$, we find its corresponding node *v* in the spectrum graph. In Step 6 of the RSGM algorithm, the length of the text string *x* is a constant. In addition, the set *B*_*i*_ is stored as a hash table, and the number of operations to check if *u*^′^ exists in *B*_*i*_ is a constant. As a result, the time complexity of one search in Step 6 is *O*(1). The total number of searches starting from *u* is bounded by the out-degree of *v* multiplied by the largest size of *C*(*e*) for all edges *e* whose head node is *v*. That is, the number of searches of *u* is bounded by *dN*. Because $\left |\cup _{i=0}^{m} B_{i}\right |$ is *O*(*n*^*k*^), the total number of searches in the algorithm is *O*(*dNn*^*k*^). As a result, the time complexity of the algorithm is *O*(*dNn*^*k*^). □

Theorem 1 given the time complexity of the graph query steps in the RSGM algorithm when 2*rd* is no larger than the size of the alphabet *Σ*. In practice, the default value of the parameter *α* (the maximum difference between the masses corresponding to two nodes connected by an edge) is 350 Da and the scale factor is 100. In this case, the value of *r* is about 2.91. In addition, the out-degree of a node *v* in *G* is restricted to be at most 3 by ranking all the edges with the head node *v* in the increasing order with respect to the labeled masses and keeping only the top 3 ones. Because the maximum out-degree of the nodes in *G* is 3, the condition 2*rd*<*g* (2*rd*<18<*g*=19) is satisfied. In the experiments, the maximum out-degree of nodes in spectrum graphs was set to 3.

When 2*rd*≥*g*, the number of the suffix tree nodes searched by the algorithm is *O*(*n*), that is, the number of searched nodes is bounded by the size of the suffix tree. Each suffix tree node *u* corresponds to a spectrum graph node *v*. The total number of searches starting from *u* is bounded by the out-degree of *v* multiplied by the largest size of *C*(*e*) for all edges *e* whose head node is *v*. Therefore, the number of searches starting from *u* is bounded by *dN*, and the time complexity of the algorithm is *O*(*dNn*).

The RSGM algorithm gives all substrings in *S* that match a path from *v*_0_ to *v*_*i*_ by reporting the suffix tree nodes in *B*_*i*_. That is, the algorithm reports all substrings in *S* that match a path starting from *v*_0_. We execute the RSGM algorithm *m*−1 rounds to solve the SGM problem. In the *i*th round, the start node is set to *v*_*i*_ and the end node to *v*_*m*_. All matched substrings in the *m*−1 rounds are combined as the solution to the SGM problem.

In practice, we are interested in finding only text strings that match a long path in *G*. Let *V*_*s*_ be the set of nodes *v* in *G* such that the sum of the labeled masses on the shortest path from *v*_0_ to *v* is no larger than *β*, where *β* is a user-specified parameter. Let *V*_*t*_ be the set of nodes *v* in *G* such that the sum of the labeled masses on the shortest path from *v* to *v*_*m*_ is no larger than *β*. We only report text strings that match a path from a node in *V*_*s*_ to a node in *V*_*t*_.

### Protein sequence filtration

The spectrum graph of a top-down MS/MS spectrum usually consists of several disconnected components because the spectrum often has low fragment coverage. Based on this observation, we propose to extract *γ* mass intervals (subspectra) that are abundant with fragment masses from a query spectrum and construct a spectrum graph for each extracted mass interval, where *γ* is a user-specified parameter. In practice, the value of *γ* is chosen between 1 and 20. The spectrum graphs are searched against the protein database separately and the search results are combined to find the best sequence candidates. The filtering algorithm is called the SGM filtering algorithm.

In mass interval selection, mass intervals with the same width *δ* are reported and the width *δ* is a user-specified parameter. In practice, the value of *δ* is chosen between 500 and 1400 Da so that each reported mass interval corresponds to a sequence tag with 5−14 amino acids. (See “Parameter settings” section) The score of a mass interval is defined as the number of masses in it. The overlapping ratio of two intervals [*a*,*a*+*δ*] and [*b*,*b*+*δ*] with *a*<*b*<*a*+*δ* is defined as the ratio between the width of the overlapping region [*b*,*a*+*δ*] and the interval width *δ*, that is, $\frac {a+\delta -b}{\delta }$. If *a*<*a*+*δ*<*b*, the overlapping ratio is 0. A greedy approach is employed to find multiple top-scoring mass intervals. First, the top-scoring mass interval of the spectrum is reported. Second, we remove all mass intervals that have an overlapping ratio ≥*ρ* with the reported one, where *ρ* is a user-specified parameter, and then report the best scoring one from the remaining mass intervals. The second step is performed iteratively until a total of *γ* mass intervals are reported or no candidate mass intervals can be reported. In addition, only mass intervals with at least 6 fragment masses are reported.

We use reversed mass intervals to find text strings that match suffix fragment masses in the query spectrum. Let *b*_1_,*b*_2_,…,*b*_*k*_ be the masses in a mass interval [*m*_*l*_,*m*_*r*_] extracted from a collision-induced dissociation (CID) MS/MS spectrum with a precursor mass *M*. We generate a reversed mass interval as follows: (a) the mass interval [*m*_*l*_,*m*_*r*_] is converted into [*M*−*m*_*r*_,*M*−*m*_*l*_], and (b) for each mass *b*_*i*_, 1≤*i*≤*k*, a reversed mass *M*−*b*_*i*_ is added to the reversed mass list. For example, the reversed mass interval of a mass interval [100,400] with masses 114,213 and a precursor mass 644 is [244,544] with masses 431,530. Each extracted mass interval is further reversed to obtain a reversed mass interval in which suffix fragment masses are converted into prefix fragment masses. We search the spectrum graphs generated from the extracted and reversed mass intervals against the protein database represented by a suffix tree.

We describe three functions for assigning scores to fragment masses. For a fragment mass (*x*,*h*) with a mass value *x* and its intensity *h* in a query spectrum, the first function assigns the score 1 to the mass, that is, score(*x*,*h*)=1. The second function uses the logarithm function to normalize mass intensities to compute scores of masses. We find the lowest mass intensity *b* in the query spectrum, the score for the mass (*x*,*h*) is computed as $\text {score}(x,h) = \log _{2} \left (\frac {2h}{b}\right)$. The constant number 2 in the function is to guarantee that all scores are positive. The third function is based on the rankings of mass intensities. The fragment masses in the query spectrum are sorted in the increasing order of their corresponding intensities. Let *i* be the rank of the mass (*x*,*h*). The score of the mass (*x*,*h*) is defined as score(*x*,*h*)=1+*i*/*k* where *k* is the total number of fragment masses in the spectrum. The score of the lowest intensity mass is about 1 and the score of the highest intensity mass is 2. The three scoring functions are called the mass count score, log intensity score, and rank score, respectively. The score of a node in a spectrum graph is assigned as the score of its corresponding fragment mass.

Here we introduce a similarity score between a query spectrum and the protein sequence. A protein sequence matches a blocked pattern *P* if its text string representation contains a substring that matches *P*. The score of the pattern *P* is the number of the nodes in the corresponding path in the spectrum graph. The similarity score between a protein sequence and a spectrum graph is the score of the best scoring pattern extracted from the graph that matches the protein sequence. Let *G*_1_,*G*_2_,…,*G*_*k*_ be the set of spectrum graphs extracted from a query spectrum, the similarity score between a protein sequence and the query spectrum is the best similarity score between the protein sequence and *G*_1_,*G*_2_,…,*G*_*k*_.

After finding the best scoring pattern from *G*_1_, *G*_2_,…,*G*_*k*_ that matches a substring of the protein sequence, we compute the mass shift between the experimental fragment masses in the spectrum and the theoretical fragment masses of the protein sequence. By shifting all theoretical masses by the mass shift, we recompute the similarity score between the experimental masses and shifted theoretical ones. The new score is called the *extended similarity score* between the protein sequence and the query spectrum, which is used to rank and filter protein sequences.

## Results

Java was used to implement the SGM filtering algorithm. A 64-bit Linux desktop PC with an Intel 3.4 GHz CPU and 16 GB memory was the platform for testing all algorithms.

### Data sets

The SGM filtering algorithm was evaluated on two data sets. The first is a top-down MS data set with 2027 CID and 2027 electron-transfer dissociation (ETD) MS/MS spectra from *Escherichia coli* (EC) K-12 MG1655. The EC sample was analyzed by a liquid chromatography system coupled with an LTQ Orbitrap Velos mass spectrometer. MS and MS/MS spectra were collected at a 60000 resolution and the top 4 ions in each MS spectrum were selected for MS/MS analysis.

The second top-down MS data set was generated from MCF-7 cells. MCF-7 cells were obtained from American Tissue Culture Collection (ATCC) and maintained in minimum essential media with 5% charcoal-dextran treated fetal calf serum. In the MS experiment, soluble MCF-7 intact proteins were separated using the bead-beating based cell lysis approach followed by a filter-based desalting step. A liquid chromatograph system with a home-made long column was directly coupled with an LTQ Orbitrap Velos Pro mass spectrometer with a customized ion source. A total of 5309 CID MS/MS spectra were collected at a 60000 resolution.

### Protein spectrum-matches for evaluation

The EC raw data were centroided using msconvert in ProteoWizard [28] and deconvoluted using MS-Deconv [24]. The *Escherichia coli* K-12 MG1655 proteome database (September 12, 2016 version, 4306 entries) was downloaded from the UniProt database [14] and was concatenated with a shuffled decoy database of the same size. TopPIC [5] was employed to search the deconvoluted spectra against the target-decoy database. In TopPIC, unexpected modifications were not allowed in identified proteoforms, and the error tolerances for precursor and fragment masses were set to 15 part per million (ppm). With a 1% spectrum-level false discovery rate (FDR), a total of 1866 proteoform spectrum-matches were identified. Because of the stringent FDR cutoff, we assume that all the matches are correct in the following analysis. The 1866 proteoform spectrum-matches are referred to as the EC evaluation data set.

The SGM filtering algorithm was employed to search the spectrum in each of the 1866 matches against the EC proteome database and report 20 top scoring proteins. If the top 20 proteins contain the protein in the proteoform spectrum-match reported by TopPIC, we say the filtering is efficient. The *filtration efficiency rate* of the filtering algorithm is defined as the ratio between the number of efficiently filtered spectra and the number of all spectra in the experiment.

### Parameter settings

The EC evaluation data set was used to test the performance of the SGM filtering algorithm with various settings of the parameters (Additional file 1). In the experiments, the mass count score for fragment masses was used, only one spectrum graph was extracted from each query spectrum (*γ*=1), and reversed mass intervals were not included.

**Fig. 2:**
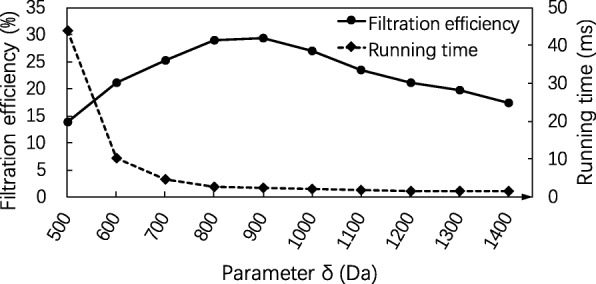
Influence of the parameter *δ* on the SGM filtering algorithm. The filtration efficiency and running time of the SGM filtering algorithm are compared on the EC evaluation data set with various settings of the parameter *δ* from 500 to 1400 Da and fixed parameter settings *α*=300 Da, *β*=200 Da, *λ*=*∞* (no masses are removed in the spectral preprocessing), and the error tolerance *ε*=0.02 Da

We first evaluated the filtration efficiency with various settings of the three parameters *α*, *β* and *δ*. In the filtering algorithm, no masses are removed in the spectral preprocessing (*λ*=*∞*), the error tolerance *ε* was set to 0.02 Da. To test the 3 parameters, we fixed the settings of 2 parameters and compared the performances with various settings of the third parameter. First, the parameters *α* and *β* were set to *α*=300 Da and *β*=200 Da, and the parameter *δ* was set to various values between 500 to 1400 Da. The algorithm obtained the best filtration efficient rate (29.21*%*) when *δ*=900 Da (Fig. 2). Although the running time decreases as the value of *δ* increases, there is no significant difference in the running time when *δ*≥900 Da.

**Fig. 3:**
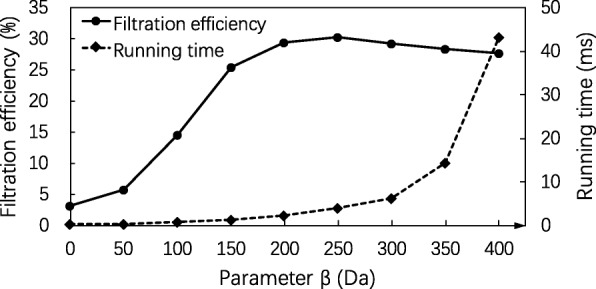
Influence of the parameter *β* on the SGM filtering algorithm. The filtration efficiency and running time of the SGM filtering algorithm are compared on the EC evaluation data set with various settings of parameter *β* from 0 to 400 Da and fixed parameter settings *δ*=900, *α*=300, *λ*=*∞* (no masses are removed in the spectral preprocessing), and the error tolerance *ε*=0.02 Da

**Fig. 4:**
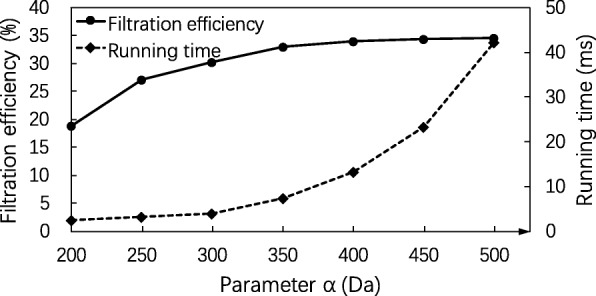
Influence of the parameter *α* on the SGM filtering algorithm. The filtration efficiency and running time of the SGM filtering algorithm are compared on the EC evaluation data set with various settings of parameter *α* from 200 to 500 Da and fixed parameter settings *δ*=900, *β*=250, *λ*=*∞* (no masses are removed in the spectral preprocessing), and the error tolerance *ε*=0.02 Da

Similarly, we evaluated the performance of the SGM filtering algorithm with various settings of *β* between 0 and 400 Da and fixed settings *δ*=900 Da and *α*=300 Da (Fig. 3) and that with various settings of *α* between 200 and 500 Da and fixed settings *δ*=900 Da and *β*=250 Da (Fig. 4). The algorithm achieved the best filtration efficiency when *β*=250 Da in the first experiment and when *α*=350 Da in the second experiment. The best filtration efficiency rate 33.07*%* was obtained when *α*=350 Da, *β*=250 Da and *δ*=900 Da.

In practice, the error tolerance *ε* in the SGM filtering algorithm is determined by the precision of the mass spectrometer. By using the following parameter settings: *α*=350 Da, *β*=250 Da, *δ*=900 Da, *λ*=*∞*, we compared the performances of the filtering algorithm on the EC evaluation data set with various settings of the error tolerance *ε* in [0,0.05] Da. The best filtration efficient rate was achieved when *ε*=0.02 Da (Table 2).

**Table 2:** The filtration efficiency and running time of the SGM filtering algorithm with various settings of the error tolerance *ε* from 0 to 0.05 Da and fixed parameter settings *α*=350, *β*=250, *δ*=900, and *λ*=*∞* are compared on the EC data set

Error tolerance (Da)	Filtration efficiency	Running time (ms)
0	8.04%	1.64
0.01	31.14%	4.81
0.02	33.07%	7.87
0.03	31.30%	14.03
0.04	29.47%	20.62
0.05	28.19%	25.58

We also tested how the parameter *λ* in spectral preprocessing affects the performance of the SGM filtering algorithm. We set *α*=350 Da, *β*=250 Da, *δ*=900 Da, *ε*=0.02 Da, and compared the performances of the filtering algorithm on the EC evaluation data set with various settings of the parameter *λ* between 2 and 12. By setting *λ*=8, we achieved the best filtration efficiency (Table 3). Based on the experimental results, the default settings of the parameters *α*, *β*, *δ*, *ε*, and *λ* are given in the Additional file 1.

**Table 3:** The filtration efficiency and running time of the SGM filtering algorithm with various settings of the parameter *λ* from 2 to 12 Da and fixed parameter settings *α*=350, *β*=250, *δ*=900, and *ε*=0.02 Da are compared on the EC data set

*λ*	Filtration efficiency	Running time (ms)
3	25.51%	1.98
4	29.64%	3.28
5	31.94%	4.19
6	33.82%	5.04
7	34.03%	5.60
8	34.35%	6.01
9	33.82%	6.55
10	33.60%	6.65
11	33.55%	7.17
12	33.55%	7.57
No reserve	33.07%	7.87

#### Multiple spectrum graphs

The methods described in “Protein sequence filtration” section was used to extract multiple spectrum graphs with two parameters: the number *γ* of extracted spectrum graphs and the maximum overlapping ratio *ρ* between two mass intervals. We compared the performances of the SGM filtering algorithm with various settings of the two parameters: *γ*=1,3,5,10,20, and *ρ*=0*%*,20*%*,50*%*,80*%*. Other parameters were set to the default values. By using the parameter settings *γ*=20 and *ρ*=50*%*, the best filtration efficiency was obtained (Table 4), but the running time with the setting was about 7 times longer than that with the setting *γ*=20 *ρ*=20*%* (Table 5). Because the numbers of fragment masses in many query spectra are limited, the total number of spectrum graphs reported from a query spectrum often depends on the maximum allowed overlapping ratio. When the maximum overlapping ratio is 0%, that is, only a few no overlapped spectrum graphs are generated in most cases. When the maximum overlapping ratio is 80%, more spectrum graphs are generated, significantly increasing the running time. This is the reason why the parameter setting *γ*=20 and *ρ*=20*%* achieved a good balance between the filtration efficiency and speed.

**Table 4:** Filtration efficiency rates of the SGM filtering algorithm with various settings of the parameters *γ* and *ρ* on the EC evaluation data set

*ρ*	0%	20%	50%	80%
*γ*				
1	34.35%	34.35%	34.35%	34.35%
3	48.23%	47.91%	47.96%	44.53%
5	51.93%	51.82%	52.57%	49.73%
10	53.84%	54.13%	56.48%	54.45%
20	53.48%	54.44%	57.88%	57.88%

**Table 5:** The average running time in millisecond (ms) per spectrum of the SGM filtering algorithm with various settings of the parameters *γ* and *ρ* on the EC evaluation data set

*ρ*	0%	20%	50%	80%
*γ*				
1	6.01	6.01	6.01	6.01
3	14.08	15.66	22.29	19.55
5	21.88	31.82	62.63	37.91
10	32.06	63.18	234.41	135.17
20	33.15	76.41	508.78	557.72

#### Reversed mass intervals

To evaluate the performance of the SGM algorithm with reverse mass intervals. The spectrum graphs generated from reversed mass intervals were used to improve the filtration efficiency. When *γ*=20 and *ρ*=20*%*, experiments showed the filtration efficiency rate (71.01*%*) of the SGM filtering algorithm with reversed mass intervals was much higher than that (54.44*%*) without reversed mass intervals.

#### Scoring functions

Using the parameter settings in Additional file 1, we compared the performances of the SGM filtering algorithm with three scoring functions: the mass count score, the log intensity score, and the rank score, on the EC evaluation data set. The mass count score and the rank score obtained the same filtration efficiency rate 71.01*%*, which was higher than that of the log intensity score (65.38*%*). When the query spectrum contains several very high intensity fragment masses, many random text strings that match these masses may have high scores and the target protein may be missed in the reported top proteins. This might be the reason that using the log intensity score does not achieve good filtration efficiency. In addition, the extended similarity score (see “Protein sequence filtration” section) based on mass count scores further improved the filtration efficiency rate to 76.63*%*. Based on the experimental results, we use the extended mass count score as the default scoring function in the SGM filtering algorithm.

### Comparison with other filtering algorithms

Two tag-based algorithms for protein sequence filtration were compared with the SGM filtering algorithm on the MCF-7 data set. The first tag-based algorithm is implemented in MS-Align+Tag (http://bioinf.spbau.ru/proteomics/ms-align-plus-tag) and referred to as TAG-1; the second tag-based algorithm is implemented in MSPathFinder [29] and referred to as TAG-2. The two tag-based algorithms were implemented in C++. The MCF-7 raw data was centroided using msconvert in ProteoWizard [28] and deconvoluted using MS-Deconv [24]. The human proteome database (July 9, 2016 version, 20191 entries) was downloaded from the UniProt database and concatenated with a shuffled decoy database with the same size.

The SGM filtering algorithm was coupled with the spectral alignment algorithm in TopPIC for spectral identification. The coupled method consists of four steps: (1) the SGM filtering algorithm reports 20 top scoring protein sequences for each query spectrum, (2) the 20 protein sequences are aligned with the query spectrum to report the best scoring proteoform spectrum-match, (3) an E-value of the best scoring match is computed, and (4) the target-decoy approach [30] is used to estimate FDRs and filter identified proteoform spectrum-matches with a 1% spectrum-level FDR. Similarly, the two tag-based algorithms were coupled with the spectral alignment for spectral identification. The three filtering algorithms were compared on the number of proteoform spectrum-matches identified from the MCF-7 data set.

**Fig. 5:**
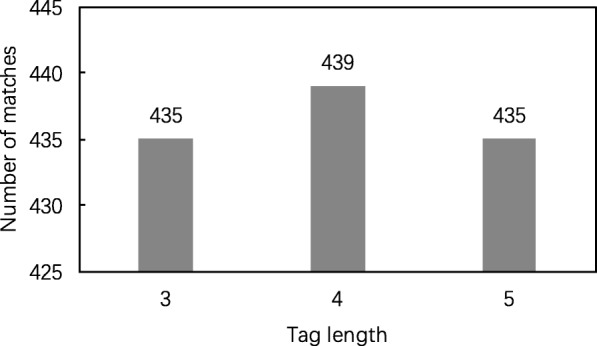
Comparison of the performances TAG-1 with various settings of the tag length *l*. With a 1% spectrum level FDR, the numbers of proteoform spectrum-matches identified by the TAG-1 filtering algorithm with various settings of the tag length *l* are compared on the MCF-7 data set. The TAG-1 filtering algorithm is coupled with the spectral alignment algorithm in TopPIC for spectral identification

Both TAG-1 and TAG-2 use spectrum graphs without gaps to obtain sequence tags. TAG-1 first finds the longest path in a spectrum graph and then generates all substrings with a fixed length *l* of the longest path as sequence tags. TAG-2 generates all possible sequence tags with a length in [*l*_*min*_,*l*_*max*_] from a spectrum graph, where *l*_*min*_ is a minimum length and *l*_*max*_ is a maximum length. To find the best parameter settings, we compared the performances of TAG-1 with various settings *l*=3,4,5 and those of TAG-2 with various settings of the parameters *l*_*min*_=3,4,5,6 and *l*_*max*_=7,8,9. The error tolerance for matching a mass difference to an amino acid residue in spectrum graph generation was set as 15 ppm. Experimental results showed that the TAG-1 algorithm reported the largest number of identifications when *l*=4 (Fig. 5) and that the TAG-2 algorithm achieved the best performance when *l*_*min*_=4 and *l*_*max*_=9 (Table 6). These parameter settings in the tag-based algorithms were used in the following comparison. The parameter settings of the SGM filtering algorithm are given in Additional file 1. The parameter setting of TopPIC were the same as those in Additional file 2 except that at most 1 unexpected mass shift was allowed in an identified proteoform.

**Table 6:** The numbers of proteoform spectrum-matches identified from the MCF-7 data set by TAG-2 filtering algorithm with various settings of the minimum tag length *l*_*min*_ and the maximum tag length *l*_*max*_

*l*_*max*_	7	8	9
*l*_*min*_			
3	498	506	514
4	593	594	597
5	580	578	583
6	461	460	459

The TAG-2 filtering algorithm is coupled with the spectral alignment algorithm in TopPIC for spectral identification

**Fig. 6:**
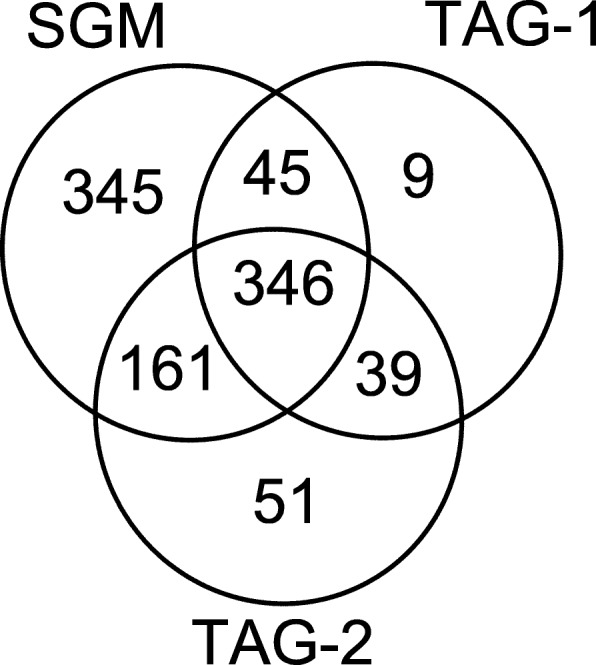
Comparison of the numbers of identifications of SGM, TAG-1, and TAG-2. With a 1% spectrum-level FDR, the numbers of proteoform spectrum-matches identified by the SGM, TAG-1, and TAG-2 filtering algorithms are compared on the MCF-7 data set. The three algorithms are coupled with spectral alignment algorithm in TopPIC for spectral identification

The average running time (540 ms) of the SGM algorithm for a query spectrum was about 2 times of the tag-based algorithms (TAG-1: 250 ms; TAG-2: 166 ms). Because the SGM algorithm has a larger search space compared with the tag-based algorithms, it is reasonable that its running time is longer than TAG-1 and TAG-2. With a 1% spectrum-level FDR, the SGM, TAG-1, TAG-2 filtering algorithms identified 897, 439, and 597 proteoform spectrum-matches, respectively. A total of 346 spectra were identified by all the three methods. TAG-1 missed 506 spectra and TAG-2 missed 390 spectra which were identified by the SGM method (Fig. 6), showing that the filtration efficiency of the SGM algorithm is much better than the two tag-based algorithms.

## Discussion

Many software tools for top-down mass spectral identification use protein sequence filtering methods based on sequence tags or gapped tags. But these methods often fail to achieve high sensitivity because of missing peaks and noise peaks in spectra. The proposed SGM filtering method provides to a solution to the problem of noise peaks by skipping the step of determining which peaks are signal ones. Compared with the methods based on gapped tags, the SGM filtering method includes all possible gapped tags in tag-sequence matching, increasing the sensitivity of protein sequence filtration. Although the SGM filtering algorithm is more complex than the blocked pattern match algorithm, its speed is still acceptable. The main reason is that high accuracy fragment masses efficiently filter out most candidate substrings in the intermediate steps in the search.

The SGM filtering method can be combined with other methods to further improve the performance of protein sequence filtration. For example, a tag-based method is used as the first filtering method for spectral identification, and only spectra that are not identified in the first found are searched by the SGM filtering method. Because the tag-based method is fast and the SGM filtering method is sensitive, the combined approach is capable of achieving both high speed and high sensitivity.

The SGM filtering method still has some limitations. First, it often reports many candidate proteins for spectra with many noise peaks. As a result, the target protein may be missed when only a limited number of proteins are reported as filtering results. Second, the three scoring functions used in the SGM filtering method are not powerful in distinguishing correct matches between spectra and protein sequences from incorrect ones. Advanced machine learning methods are needed to design a good scoring function.

## Conclusions

In this paper, we proposed an efficient spectrum graph-based filtering algorithm for top-down mass spectral identification and tested the algorithm on two real top-down MS data sets. Compared with tag-based methods, the SGM filtering algorithm circumvents the steps of tag generation and directly searches spectrum graph against the protein database, simplifying data processing and increasing filtration efficiency. The experimental results on the real data demonstrate that the SGM filtering algorithm outperforms the two tag-based algorithms in filtration efficiency.

## Additional files

Additional file 1 A summary of parameters and their default values of the SGM filtering algorithm. (PDF 28 kb)

Additional file 2 The parameter settings of TopPIC used in the experiments. (PDF 13 kb)

## Abbreviations

ATCC: American tissue culture collection
CID: Collision-induced dissociation
Da: Dalton
EC: Escherichia coli
ETD: Electron-transfer dissociation
FDR: False discovery rate
LTQ: Linear trap quadrupole
MCF-7: Michigan cancer foundation-7
MS: Mass spectrometry
MS/MS: Tandem mass spectra
ppm: Part per million
PTM: Post-translational modification
RSGM: Restricted spectrum graph matching
SGM: Spectrum graph matching
