# Supplementary material for: A graph-based filtering method for top-down mass spectral identification
Source: BMC Genomics. 2018 Sep 24;19(Suppl 7):666. doi: 10.1186/s12864-018-5026-x (PMC6157290; doi:10.1186/s12864-018-5026-x)
Supplement: Supplementary file 1 — A summary of parameters and their default values of the SGM filtering algorithm. (PDF 28 kb) [file 12864_2018_5026_MOESM1_ESM.pdf]

Parameter	Description	Default value
α	The maximum difference between two masses whose corresponding nodes in the spectrum graph are connected by an edge	350 Da
β	The maximum difference between the masses of the node v_0 and the start node s, and the maximum difference between the masses of the end node t and the node v_m	250 Da
δ	The width of mass intervals extracted from the query spectrum	900 Da
γ	The maximum number of mass intervals extracted from the query spectrum	20
ρ	The maximum overlapping region between two mass intervals extracted from the query spectrum	20%
λ	A mass x is kept in the preprocessing only if its intensity is in the top λ among all masses in the interval $[x - 100, x + 100]$.	8
ϵ	The error tolerance for matching a mass in a blocked pattern to the mass of a text string	0.02 Da
