# Supplementary material for: A graph-based filtering method for top-down mass spectral identification
Source: BMC Genomics. 2018 Sep 24;19(Suppl 7):666. doi: 10.1186/s12864-018-5026-x (PMC6157290; doi:10.1186/s12864-018-5026-x)
Supplement: Supplementary file 2 — The parameter settings of TopPIC used in the experiments. (PDF 13 kb) [file 12864_2018_5026_MOESM2_ESM.pdf]

Parameter	Value
Number of combined spectra	1
Fragmentation method	FILE
Search type	TARGET+DECOY
Fixed modifications	None
Maximum number of unexpected modifications	0
Error tolerance	15 ppm
Cutoff type	FDR
Cutoff value	0.01
Allowed N-terminal forms	NONE,NME,NME_ACETYLATION
Maximum mass shift of modifications	500 Da
Thread number	1
E-value computation	Lookup table
